# Predation strongly limits demography of a keystone migratory herbivore in a recovering transfrontier ecosystem

**DOI:** 10.1002/ece3.9414

**Published:** 2022-10-17

**Authors:** Fred Watson, Matthew S. Becker, Daan Smit, Egil Droge, Teddy Mukula, Sandra Martens, Shadrach Mwaba, David Christianson, Scott Creel, Angela Brennan, Jassiel M'soka, Angela Gaylard, Chuma Simukonda, Moses Nyirenda, Bridget Mayani

**Affiliations:** ^1^ California State University Monterey Bay Seaside California USA; ^2^ Zambian Carnivore Programme Mfuwe Zambia; ^3^ Conservation Biology and Ecology Program, Department of Ecology Montana State University Bozeman Montana USA; ^4^ Wildlife Conservation Research Unit, The Recanati‐Kaplan Centre, Department of Zoology University of Oxford Oxford UK; ^5^ African Parks Zambia, Liuwa Plain National Park Kalabo Zambia; ^6^ Worldwide Fund for Nature Lusaka Zambia; ^7^ Department of Ecosystem Science and Management University of Wyoming Laramie Wyoming USA; ^8^ Institutionen för Vilt, Fisk och Miljö, Sveriges Lantbruksuniversitet Umeå Sweden; ^9^ World Wildlife Fund Washington District of Columbia USA; ^10^ U.S. Agency for International Development Lusaka Zambia; ^11^ Zambia Department of National Parks and Wildlife Chilanga Zambia

**Keywords:** demography, migration, population, predator–prey, survival, wildebeest, Zambia

## Abstract

Large herbivore migrations are imperiled globally; however the factors limiting a population across its migratory range are typically poorly understood. Zambia's Greater Liuwa Ecosystem (GLE) contains one of the largest remaining blue wildebeest (*Connochaetes taurinus taurinus*) migrations, yet the population structure, vital rates, and limiting factors are virtually unknown. We conducted a long‐term demographic study of GLE wildebeest from 2012 to 2019 of 107 collared adult females and their calves, 7352 herd observations, 12 aerial population surveys, and concurrent carnivore studies. We applied methods of vital rate estimation and survival analysis within a Bayesian estimation framework. From herd composition observations, we estimated rates of fecundity, first‐year survival, and recruitment as 68%, 56%, and 38% respectively, with pronounced interannual variation. Similar rates were estimated from calf‐detections with collared cows. Adult survival rates declined steadily from 91% at age 2 years to 61% at age 10 years thereafter dropping more sharply to 2% at age 16 years. Predation, particularly by spotted hyena, was the predominant cause of death for all wildebeest ages and focused on older animals. Starvation only accounted for 0.8% of all unbiased known natural causes of death. Mortality risk differed substantially between wet and dry season ranges, reflecting strong spatio‐temporal differences in habitat and predator densities. There was substantial evidence that mortality risk to adults was 27% higher in the wet season, and strong evidence that it was 45% higher in the migratory range where predator density was highest. The estimated vital rates were internally consistent, predicting a stable population trajectory consistent with aerial estimates. From essentially zero knowledge of GLE wildebeest dynamics, this work provides vital rates, age structure, limiting factors, and a plausible mechanism for the migratory tendency, and a robust model‐based foundation to evaluate the effects of potential restrictions in migratory range, climate change, predator–prey dynamics, and poaching.

## Graphical abstract

The paper provides the first quantitative characterization of the demography of the blue wildebeest population in western Zambia ‐ one of Africa’s largest remaining migrations. Predation was the dominant cause of mortality, and mortality risk varied with position along the migratory route. This highlights the importance of protecting the migratory range of large herbivores.

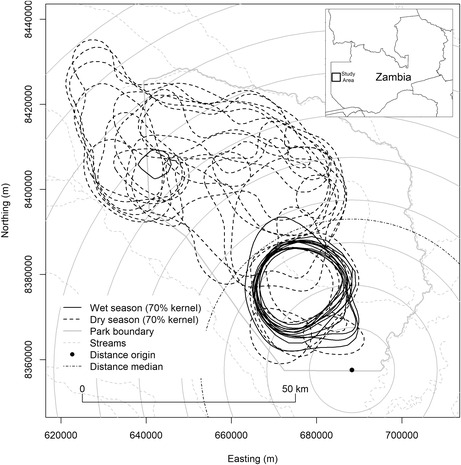

## 1 INTRODUCTION

Large ungulates play a critical role in the functioning of ecosystems through their impacts on vegetation structure, diversity, and nutrient cycling, and as prey for top carnivores, yet they are some of the most imperiled species worldwide (Ripple et al., 2015). Ungulates are most abundant in migratory populations, and seasonal migration is thought to mitigate the regulatory effects of food limitation and predation (Fryxell et al., 1988; Hopcraft et al., 2015). While previous research has focused on spatio‐temporal variation in food resources and predation, the connection between migratory behavior and population limitation is unknown for most large herbivore migrations (Bolger et al., 2008). Rapid human‐induced ecological change has made the long‐term prospects for large ungulates in general—and migratory populations in particular—very poor given that they are wide‐ranging, often predictable in their migratory routes and timing, and rarely have their entire migratory range protected (Berger, 2004; Hopcraft et al., 2015; Ripple et al., 2015). Such characteristics make these populations extremely sensitive to human impacts in the form of overhunting, poaching, human barriers such as fencing, and habitat loss from agricultural conversion and other practices (Bolger et al., 2008), particularly in the face of climate change (Durant et al., 2015). In Africa the current decline of migratory herbivores is most pronounced (Ripple et al., 2015) and exemplified by the iconic migratory ungulate, the blue wildebeest (*Connochaetes taurinus*). Once widespread throughout savannah Africa the wildebeest has experienced range‐wide declines, specifically when migratory routes have been stopped or altered, resulting in rapid population collapse (Berger, 2004; Bolger et al., 2008; Morrison et al., 2016). While these declines have been well documented, the factors driving demography of migratory wildebeest populations—and their subsequent declines—are not well understood.

Long‐lived mammals such as ungulates exhibit age and sex‐dependent vital rates (Eberhardt, 2002) and these rates can be impacted by an array of ecological factors. These broadly include resource limitation from forage and minerals as well as predation and predation risk effects (e.g., changes in behavior, habitat selection, nutrition and reproduction as a result of a predator being on the landscape, Creel et al., 2019), all of which can interact and in turn can be affected by and interact with human activities. To better conserve rapidly declining migratory populations, Bolger et al. (2008) emphasized an integrated approach in part aimed at understanding population limitation in all phases of the migratory cycle and the demographic consequences of human disruption to these migrations. Despite blue wildebeest being very well‐studied through the seminal work on the Serengeti‐Mara populations (Hopcraft et al., 2015; Mduma et al., 1999; Sinclair & Arcese, 1995a, 1995b; Sinclair & Norton‐Griffiths, 1979) few intensive, long‐term, and individual‐based demographic studies exist on this keystone species to evaluate population limitation in this context (Bolger et al., 2008). And more such long‐term demographic studies are needed on large ungulates in general (Gaillard, 2013; Schradin & Hayes, 2017).

Western Zambia's Liuwa Plain National Park (LPNP) and the surrounding area comprises the Greater Liuwa Ecosystem (GLE), which currently houses what is thought to be the second‐largest remaining wildebeest migration in Africa, and the largest population of common blue wildebeest (Estes, 2014). It is part of a larger ecosystem spanning much of northeastern Angola and comprising the Liuwa‐Mussuma Transfrontier Conservation Area (LMTFCA, M'soka et al., 2016). Historically wildebeest are thought to have seasonally migrated long distances between both countries prior to being decimated in the decades‐long Angolan civil war (1975–2002), and the boundaries of the LMTFCA were in part designed to protect the historical migration route and patterns, although this migration is largely undocumented (Harris et al., 2009). African Parks Zambia, under a public private partnership with the Department of National Parks and Wildlife (DNPW), African Parks, and the Barotse Royal Establishment, have invested substantially in ecosystem recovery through natural resource protection and economic development of LPNP and allowed for rapid recovery of wildlife populations. However, the ecological and anthropogenic factors limiting this migratory population across its range are not well understood, and this has significant implications for conservation and management of both this keystone species and ecosystem. Wildebeest are experiencing an array of limiting demographic impacts in the form of predation from a recovering carnivore population (Creel et al., 2017, 2019; Droge et al., 2017a; M'soka et al., 2016), poaching, and human encroachment, and at present no transboundary migration to Angola has been documented (Droge et al., 2019). In order to assess the factors limiting recovery of this migratory population we conducted intensive individual‐based demographic studies of radio‐collared individuals from 2012–2019 in order to (1) estimate survival and reproduction of adult females and recruitment of calves, (2) identify and evaluate factors most affecting survival, and (3) make recommendations for conservation and management of the study population and for extant or recovering large herbivore migrations in general.

## 2 METHODS

### 2.1 Study area

**FIGURE 1:**
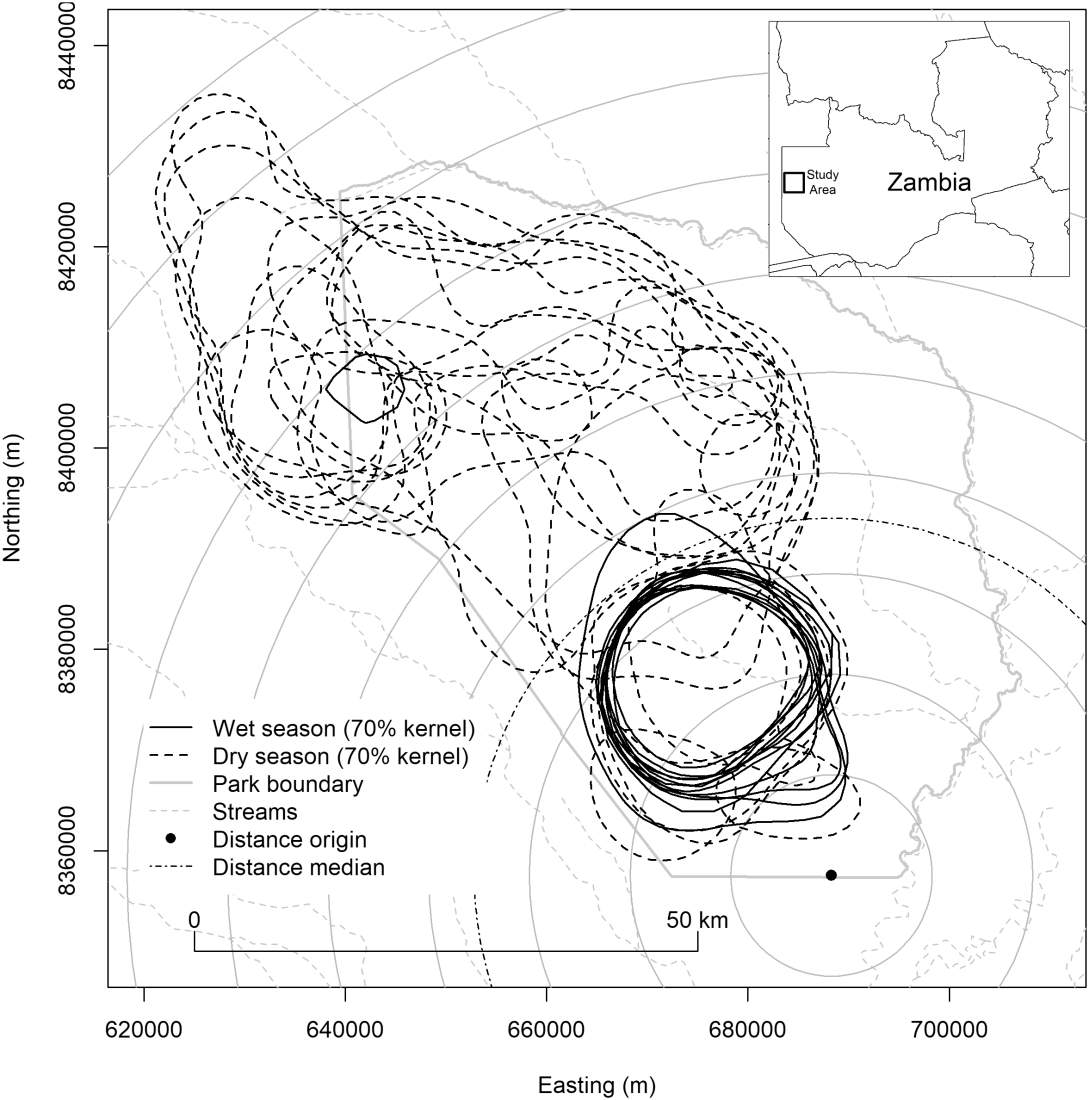
Study area in and around Liuwa plain National Park, Northwest Zambia. Concentric circles indicate distance from a point beyond the southeastern extreme of wildebeest range, as a basis for a one‐dimensional measure of location along the migratory corridor.

The LMTFCA is located on the boundary of Western Zambia and Angola, and our study area comprised the Greater Liuwa Ecosystem (GLE), consisting of LPNP and the Upper West Zambezi Game Management Area (UWZGMA, Figure 1). The LPNP covers 3660 km^2^ of seasonally flooded grassland with isolated patches of open broad‐leafed woodlands. The GLE experiences extensive rains and flooding during the wet season (Dec–May) and isolated seasonal and permanent water sources remain during the dry season (June–Nov).

Declared a National Park in 1972, LPNP has been a conservation area since the 1880s, when it was declared by the Lozi King as a royal hunting ground. Following park designation, settlements were still permitted and the human population within and around LPNP numbered approximately 17,000 people during this study (African Parks, 2018). Largely as a result of a decades‐long civil war in neighboring Angola (1975–2002) the wildlife populations in the GLE were decimated, with many species extirpated or reduced to severely low numbers (M'soka et al., 2016). In 2003, African Parks signed a 20‐year public–private partnership with the DNPW with the goal of restoring the GLE ecosystem and making it a profitable wildlife‐based economy. The GLE wildebeest population is thought to have been transboundary across the border with Angola. Currently it exists only within Zambia, migrating seasonally between a rainy season range generally in the southern portion of LPNP and a dry season range in northern LPNP and the adjacent UWZGMA (M'soka et al., 2017). Liuwa also contains an unknown but large number of oribi (*Ourebia ourebi*) and approximately 5000 migratory zebra (*Equus burchelli*), as well as smaller numbers of red lechwe (*Kobus leche leche*), tsessebe (*Damaliscus lunatus lunatus)*, reedbuck (*Redunca arundium*), common duiker (*Sylvicapra grimmia*), buffalo (*Syncerus caffer*), eland (*Tragelaphus oryx*), roan (*Hippotragus equinus*), and sitatunga (*Tragelaphus spekii*) (Viljoen, 2009, 2011, 2013). A 1970 aerial census counted 12,691 wildebeest and estimated a population as high as 16,000 (Estes & East, 2009). More recently, 10 aerial surveys between 2001 and 2018 estimated wildebeest and other herbivores, with best estimates ranging between 23,000 and 46,000 and wide confidence intervals where these were quantified (African Parks, 2018; Viljoen, 2013). Almost the entire population is located in the southeast portion of the range from December to April; migration to the northwest occurs in May and June and is complete by July. Migration to the southeast occurs in October and November. Calving occurs around the first of October, and evidence of weaning (i.e., spatial dissociation of calves from their mothers) begins around July.

### 2.2 Capture and collaring

We focused our study around adult female wildebeest and utilized radio‐collars for the basis of this work. We initiated collaring in May 2012 when a study population of 45 adult cows were fitted with Telonics VHF collars with a 5‐year battery life. Because losses to predation and poaching occurred throughout the study, we attempted to maintain a study population of 30–50 adult cows year‐round and thus conducted additional collar deployments of 6–15 animals from October to December in ensuing years, following calving when most of the population was congregated in the south. A total of 117 collars were deployed on 107 cows, including 95 VHF collars, 18 GPS collars, four satellite/GPS collars, and 10 recollarings of previously collared animals. Animals were immobilized with cartridge‐fired dart rifles projected from vehicle or helicopter in the initial capture, and then by vehicle thereafter. Immobilized wildebeest were monitored for temperature and respiration and fitted with collars while blood, tissue, hair, and fecal samples were obtained from each animal. Measurements of shoulder height and body length were performed, as well as measurements of tooth wear on the I1 and I2 incisor teeth to estimate age (Christianson et al., 2018). When possible, the animal was physically checked for pregnancy. The immobilization was then reversed, and the animal was monitored for recovery. All procedures followed guidelines established by the Department of Veterinary Services (Zambia) and the Department of National Parks and Wildlife (Zambia) and a Zambian‐registered veterinarian conducted and oversaw all operations.

### 2.3 Aging

Given that large ungulates exhibit age‐specific rates of survival and reproduction (Eberhardt, 2002), it was important to have an estimate of age for each cow. Thus, we developed a novel aging method based on arcade breadth, labio‐lingual width, and crown‐height measurements of the first incisors that correlated well with cementum annuli analyses from wildebeest that had been predated during the course of the carnivore work and their incisors removed for analyses (Christianson et al., 2018). Consequently, when wildebeest were immobilized a trained team collected these measurements on both first incisors to provide the basis for an estimate of the cow's age using an algorithm detailed in the Appendix A.

### 2.4 Field methods for demographic data collection

Beginning in September 2012 we attempted to visually locate each collared cow on the ground at least once every 2 weeks. Field teams covered the study area daily by motorbike or vehicle to radio‐track collared cows, and periodic aerial tracking flights were also utilized when possible using an ultralight aircraft (Foxbat, Aeroprakt, Ukraine). Given that the population was migratory and unstudied, it was difficult to implement a stratified random sampling procedure of collared cows. Thus, we employed an intensive search effort throughout the study area to locate every animal regularly, logging approximately 800–1100 person days in the field each year.

We not only collected survival and reproduction data on these collared cows but also used these locations to collect recruitment and composition data on the associated herds. Collecting data from herds located through radio‐tracking of collared cows minimized detection bias that might be present with opportunistically sampling any herd for composition and calf: cow ratios. Upon detection of collared cows a herd count was conducted and the animals were classified by sex and age (calves, yearlings, cows, or bulls). Sex was primarily determined using characteristics of the genital and abdominal region. Calves were not sexed. Yearling classification was not always possible near October given the similarity to subadults as the animals neared their third year. While smaller to medium‐sized herds could be counted and classified accurately and completely (i.e., a total count), in most instances large herds were substantially more difficult, if not impossible, to age and sex in their entirety. However, these herds comprised the majority of the population, often differed substantially in composition, and could have different rates of calf survival from smaller herds (Estes, 2014). Thus, they were critical to estimates of calf recruitment and sex and age composition. To address this we developed a herd sampling method based on the position of the focal collared cow in the herd. Upon visual location of the collared cow in a large herd we classified a sample of animals in the section of the herd radiating out from around the collared cow until we were unable to accurately classify individuals by sex and age, at which point the observation stopped. Herds sampled in this way averaged 685 animals, of which an average of 57 animals were classified. While some biases by age and sex can be present in sampling herds, the loosely defined aggregations typical of large herds (not under immediate predation pressure) made other methods unfeasible given that herd size and composition changed continually and spacing precluded accurate and complete sexing and aging in large groups. Using collared cow locations as the sampling commencement point provided a degree of randomization by avoiding potential systematic biases in composition resulting from an animal's peripheral or interior herd position (Estes, 2014) and allowed us to accurately record data on herd composition and calf: cow ratios. A total of 6734 total counts and sample counts (hereafter herd counts) were conducted. We retained 5440 of these for fitting the count‐based vital‐rate model (see below), excluding counts near and during calving season (1 September to 31 October) when the presence or absence of a calf with a collared cow could not be reliably determined, and excluding counts during incomplete years at the start and end of the study. This yielded 241,251 records of whether an animal was a calf or a cow (and not a yearling or bull).

We determined whether a collared cow had a calf by a composite of interactive behaviors between the animals including nursing, grooming, and spacing (following). Vital rate estimates based on the calves of collared cows were derived from 216 cow‐years observing 94 cows. We increased field efforts in three periods: (1) August–September, in order to facilitate estimation of first‐year recruitment, (2) October–November, in order to facilitate estimation of fecundity, and (3) April–May, in order to facilitate estimation of wet‐season calf survival.

We quantified causes of mortalities using three independent sources of information: (1) monitoring of collared cows, (2) opportunistic detection of mortalities within the population as a whole in the course of year‐round work on wildebeest and carnivores, and (3) hunt follows as described in Droge et al. (2017a). Cow collars were equipped with mortality sensors, which became active after 12 h of no movement. When a mortality signal was detected, the collar was located and the area was assessed for signs of predators, poaching, disease, or starvation as per prior work on predation (Creel et al., 2019, 2017; Droge et al., 2017a). Typically, given the high density of hyenas (see below), carcasses rarely had much of anything left besides the collar, making it difficult to definitively determine cause of death. Calf mortality was determined when the calf was no longer detected with the collared cow, except after July when calves and cows tend to begin dissociating as pregnant cows near parturition (Estes, 2014).

Through concurrent studies we were able to quantify the composition of the predator population, which provided more specificity about predator influences on the wildebeest population, including both predation and predation risk effects (Christianson et al., 2018; Creel, 2018; Creel et al., 2017, 2019; Droge et al., 2017a, 2017b, 2019; M'soka et al., 2016, 2017). Wildebeest are the primary prey for hyena, lion, and African wild dog, and an important prey for cheetah. Due to depletion by poaching and conflict, the GLE carnivore guild is unusual because of its low numbers of lions and dominance of spotted hyena, which outnumber all other carnivores by orders of magnitude (M'soka et al., 2016). The highest densities of spotted hyena (hereafter, hyena) occur in southern LPNP in an area overlapping the wet season wildebeest range. We intensively studied 661 individual hyenas in 6–11 clans numbering 101–289 animals annually (annual mean 195). Hyenas reside throughout the entire wildebeest range at lower densities, and some long‐distance commuting occurs between the wet and dry season wildebeest ranges (ZCP *unpublished data*). By 2003 the lion population was reduced to a single lioness, with subsequent reintroductions of two males, two females, and one male in 2009, 2011, and 2016, respectively, so that the park held 3–13 adult lions in one pride during this study (annual mean: 7.1). Small populations of African wild dogs (1–2 packs of 15–22 dogs total during this study) and cheetahs (4–25 known individuals, annual mean: 11.7) were also present, with African wild dogs absent from the intensive study area from 2015 to 2019. Cheetah were stable to increasing in numbers during the study and ranged widely throughout the GLE.

Poaching was a suspected cause of mortality for a number of collared wildebeest. We removed these animals from the analyses of vital rates to avoid complications to do with varying levels of uncertainty in knowing whether or not they were actually poached and the possible biases that poaching would introduce into the estimation of environmentally determined influences on mortality. We examined the consequences of this policy and found that it had little effect on the estimation of basic vital rates and that it led to stronger evidence for the various effects on mortality.

### 2.5 Model for survival and recruitment

We quantified wildebeest survival by fitting a single survival model to three different types of field measurements. The approach is summarized here, with mathematical details provided in the Appendix A. The model was based around Siler's (1979) 5‐parameter expression of mortality hazard (i.e., risk) as a constant modified by additional hazard components representing the immature and senescent phases of an organism's life. We incorporated additional modifications that modulated the hazard by season, location, and year. In the calf‐survival analysis, we also represented calf dissociation from their mothers as a “hazard” in the mathematical sense.

The season effect was represented as a dichotomy between wet and dry seasons, with a parameter *β*_S_ measuring the degree to which hazard is greater in the wet season. The location effect was represented as a single continuous value representing distance along the major axis of migration from an arbitrary point at the southeast limits of the migration (Figure 1), with a parameter *β*_L_ measuring the degree to which hazard is greater with distance away from the southeastern corner of the study area. Year effects were represented by a vector **β**_Y_ of parameters for each year indicating the degree to which that year was more or less hazardous than other years. Calf dissociation was represented by a parameter measuring the increase in dissociation “hazard” with time since 0.75 years of age.

### 2.6 Bayesian parameter estimation and formal inference

We estimated model parameter distributions using Bayesian Monte Carlo (MC) methods based on Metropolis sampling (Givens & Hoeting, 2005; Metropolis et al., 1953; Smith, 2007). A specific likelihood function for the relevant model parameters was developed for each data set (collared cow survival, herd counts, and calf detection). Each model run involved six chains with at least 10,000 steps and adequate burn‐in. We used a sequential Bayesian approach to unify the inference resulting from different model fits (Daniels et al., 2014; Garrard et al., 2012; Kurota et al., 2009; Michielsens et al., 2008). Different subsets of the model were fit to different data sets in sequence, with the posteriors from each fit becoming the priors for subsequent fits (see Appendix A).

Formal inference was derived directly from the estimated parameter distributions. These were interpreted using a variety of metrics—depending on the parameter in question—including expected values (EV), credible intervals (CI) based on highest posterior densities, one‐sided lower credible limits (LCL0), and probabilities of being greater than zero (Pr > 0). Each of these metrics essentially describes a probability or quantile of probability that constitutes some form of evidence that can be compared to its complement in an “evidence ratio”: ER = Pr/(1‐Pr). We used the terms “substantial”, “strong”, “very strong”, and “decisive” to interpret log10 evidence ratios (LERs) of 0.5, 1.0, 1.5, and 2.0, respectively (Kass & Raftery, 1995). Credible intervals were computed at the “strong” level of evidence, that is, 90.9% = 10÷(10 + 1). We computed the distributions of derived parameters (e.g., recruitment, life expectancy, etc.) by applying their derivation to every element in the chain and computing metrics on the result. To interpret interannual variation we computed both the range and the mean absolute difference (MAD) among all years of the interannual parameters, and we did this for every element in the chains. In the case of ratios among interannual hazard parameters, we used the geometric equivalents of the above metrics. Specifically the maximum ratio (maxR) among any vector, h, of hazards was computed as $\exp\left(\text{range}\left(\ln\left(h\right)\right)\right)$, and the mean ratio (meanR) was computed as $\exp\left(\mathrm{MAD}\left(\ln\left(h\right)\right)\right)$. For the purpose of calculation of this statistic, mean first‐year hazard h1 for any given year was computed from first year survivorship S_1_ as $h_{1}=-\ln S_{1}$, and the interannual component of adult hazard was computed as hA = exp(β_Y_).

### 2.7 Longevity and life expectancy

We estimated longevity directly from modeled survivorship as the age attained by 0.1% of females. We estimated life expectancy at birth as the integral of the survivorship function (calculated numerically because there is no closed‐form antiderivative of the Siler survivorship) (Canudas‐Romo et al., 2018).

### 2.8 Population trajectory

We examined the population trajectory both through direct aerial surveys of wildebeest, and by projection of vital rates using a demographic population model. The aerial survey data provided estimates of population size and hence an indication of whether there was evidence that the population was stable, increasing, or declining. The model projections explored the self‐consistency of the estimated vital rates, and provided an alternate perspective on the question of population trend. If the model projections predicted a population explosion or crash with a severity that was inconsistent with the aerial survey data, this would be an indication of a problem with the vital‐rate estimates or the assumptions behind them, or with the accuracy of the aerial survey estimates. Conversely, if the model projections were generally consistent with the aerial survey data, this would support the notion that the study had successfully documented several of the fundamental demographic characteristics of the population, including fecundity, recruitment, age‐dependent survival, longevity, age structure, and sensitivity to season and spatial location.

Aerial population assessments were completed 12 times since 1970 using various methods. Partial‐area surveys by incompletely documented methods were conducted in 1970, 1991, and 2001 (Estes & East, 2009; Kamweneshe et al., 2003; Tembo & Siawana, 1993). A more standardized and well‐documented Systematic Reconnaissance Flight (SRF) sample methodology was used by Viljoen in 2004, 2007, 2009, 2011, 2013, and 2015, using Jolly's II method to estimate population size and 95% confidence intervals (Viljoen, 2013). More recently, African Parks carried out total area counts (Norton‐Griffiths, 1978) in 2016, 2017, and 2018, using photogrammetry to improve the accuracy of counting large wildebeest herds (African Parks, 2018).

To explore the self‐consistency of vital rates, we estimated their consequent population trajectories using a demographic population model based on standard Leslie matrix principles. A Leslie matrix model is comprised of a vector of abundances and a transition matrix. The abundance vector represented female wildebeest abundance in each of 51 six‐month age classes, ranging from age 1 to 25 years (i.e., well above the plausible maximum). The projection matrix was populated with adult survival rates from the adult survival model runs (model CC, see Table A1) and recruitment rates computed using fecundity and calf survival rates from the herd count model runs (model HC, see Table A1) and an assumed calf sex ratio of 0.5. Recruits were placed into the 1.0‐year age class. Projection from one time‐step to the next occurred over six‐month intervals. Fecundity was assumed to be equal for all adults 2.0 years and older, and zero for yearlings. This is an approximation that maintains consistency with the implicit assumption of the herd composition analysis that only cows 2.0 years and older were reproductive. Absolute abundance values were irrelevant because the model did not incorporate density dependence. So, we initialized total abundance at an arbitrary value of 1 and referred to this as a “relative population size” in the results. Demographic population projection model simulation runs are typically initialized using either an observed age distribution or the stable age distribution (Lacy et al., 2021), the latter being the age distribution to which any standard Leslie matrix model will converge under constant vital rates, regardless of the initial distribution (Caughley, 1977; Sinclair et al., 2006). If both options were available, we would argue for use of the stable age distribution in the present situation because it represents a more neutral starting point for the exploration of the general self‐consistency of vital rates estimated over an 8‐year period—but the argument is moot because a sufficient observed age distribution was not available. An observation age distribution could potentially be estimated from herd counts and tooth measurements during collaring, but the attempt would be hampered by under‐representation of collared animals between the calf and full‐grown adult stages and no individual collaring year exhibiting a large enough sample size across all age classes. Stable age distributions have been used by others in similar simulation contexts (Curtis et al., 2015; Monson et al., 2000; Taylor et al., 2009; Wiens, 2017). We computed the stable age distribution using a 50‐year warm‐up period at the start of each model run. During the warm‐up period the total abundance was held at a constant value of 1 by dividing all values by their sum after each iteration. This was done in order to facilitate examination of convergence to stable age structure, and to avoid creating a misperception that absolute abundance was relevant to this population projection exercise. We confirmed that 50 years was sufficient time for convergence by verifying that there was effectively zero change in age distribution between the last two iterations. We could potentially have instead computed the stable age distribution as the dominant right eigenvector of an annualized transition matrix (Caughley, 1977; Sinclair et al., 2006), but since our model was biannual with vital rates dependent on season, this would have been arguably more complex than simply allowing the model to converge using a warm‐up period as has been done by others in similar situations (Grear & Elderd, 2008; Wiens, 2017). After the warm‐up, the total abundance was allowed to fluctuate for five years and the annual population growth rate (*λ*) was computed as the one fifth power of the ratio between final and initial postwarm‐up abundances. A five‐year interval was chosen because we judged this to be the longest interval before unmodeled density dependent effects would need to be incorporated, and such effects were irrelevant to the scope of the exercise to examine self‐consistency of vital rates during the study period. The population projection was repeated for 5000 model runs with model parameters sampled from the posterior distributions of the adult survival and herd‐count observational models in order to compute highest posterior density credible intervals for both *λ* and the stable age structure.

## 3 RESULTS

**FIGURE 2:**
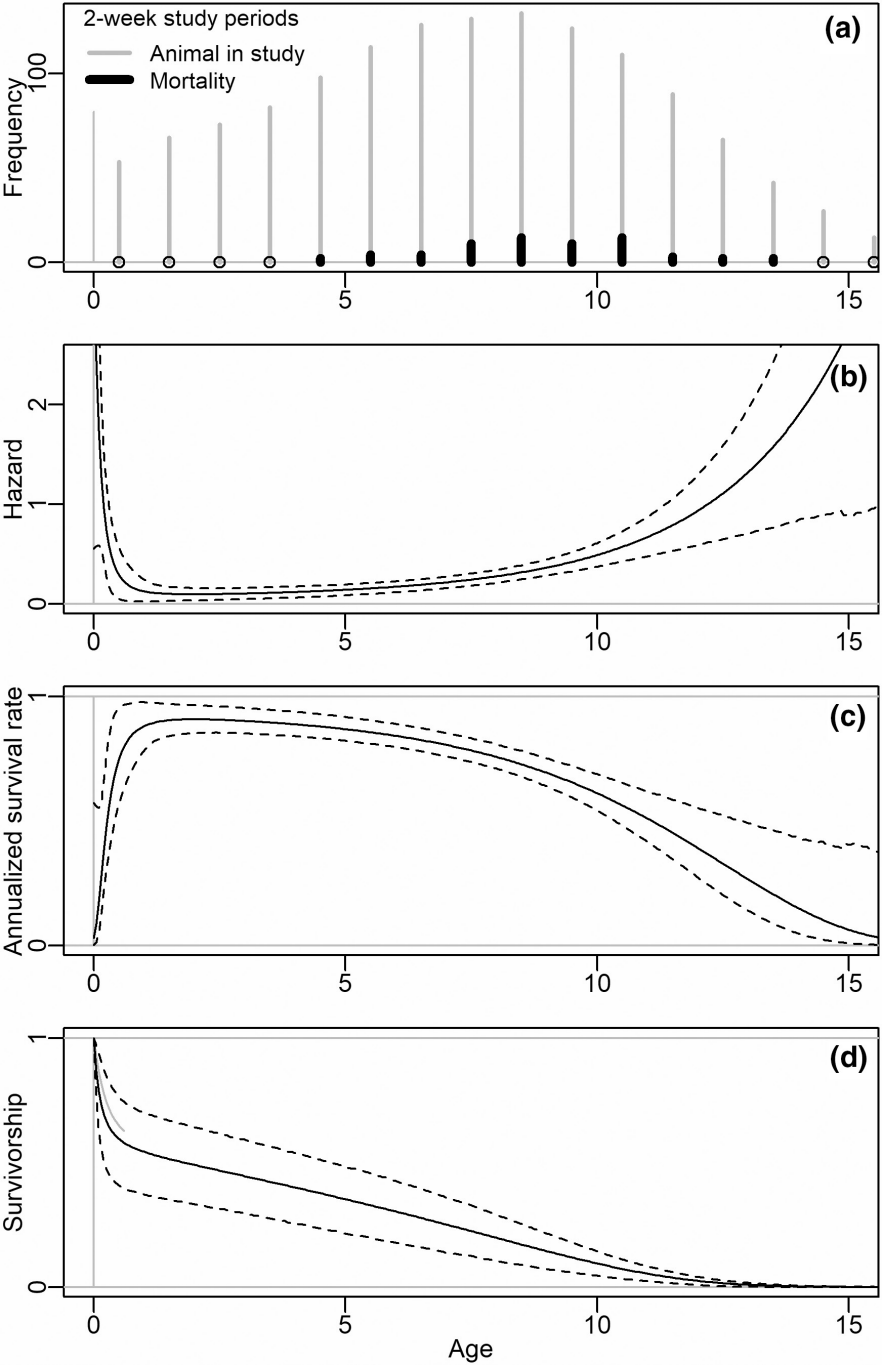
Wildebeest survival metrics from birth to senescence estimated by a Siler hazard model fitted to herd‐composition and collared‐cow data (models HC and CC): (a) frequency (number of study periods) of ages of cows at mortality (dark lines) versus being observed alive (gray lines), (b) hazard, (c) instantaneous annualized survival rate computed directly from hazard, (d) survivorship. Dashes indicate 91% credible intervals.

Results are summarized in Table A2 and Figures 2 through 6. Parameter values given below are expressed as expected values with parenthetical credible intervals or one‐sided credible limits.

**FIGURE 3:**
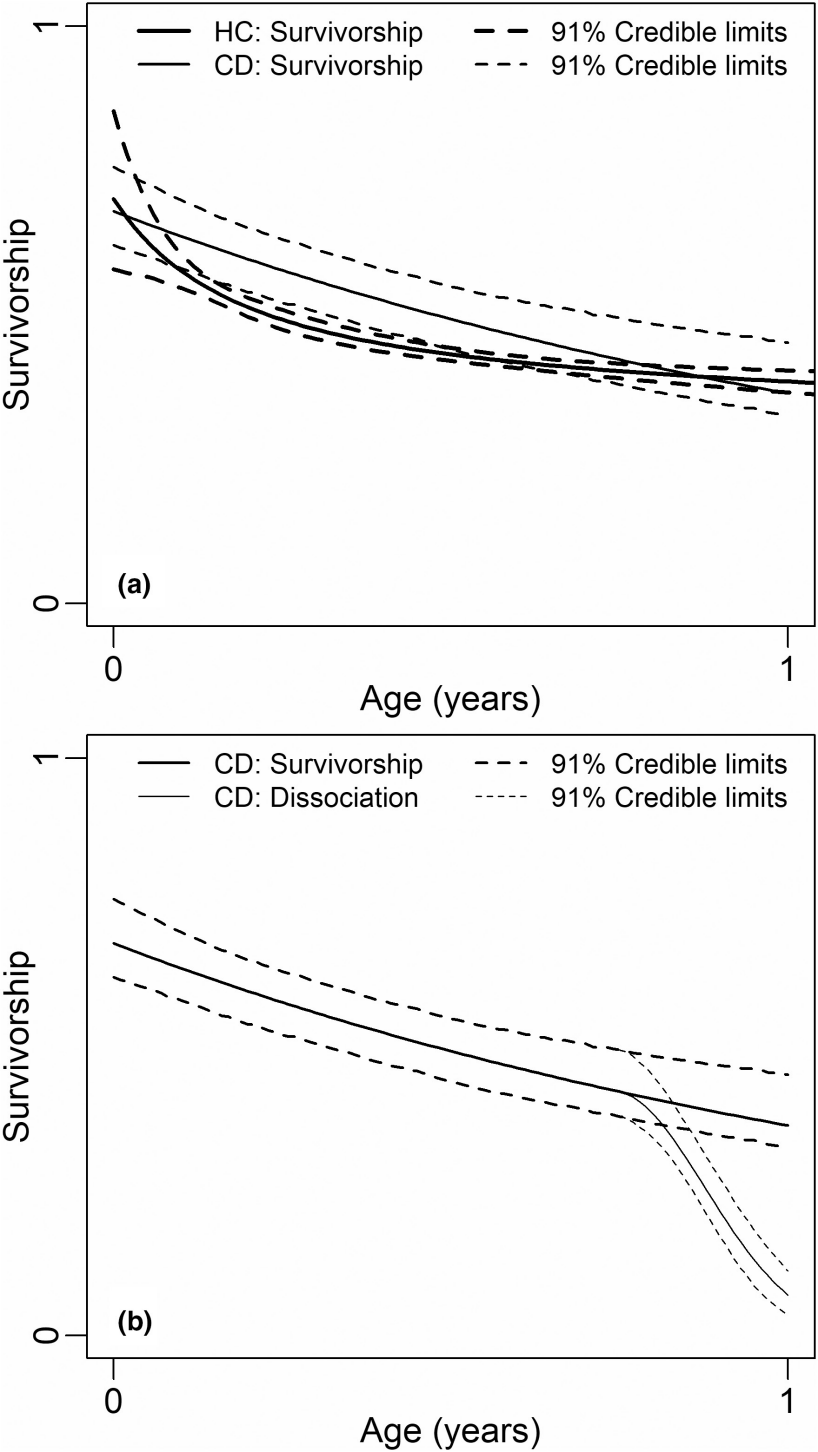
Wildebeest fecundity, survivorship, and calf dissociation from their mothers. Fecundity is plotted as the starting value for survivorship. Panel (a) compares estimates based on herd composition (HC) and collared‐cow calf detections (CD). Panel (b) illustrates calf dissociation by plotting the probability that a calf is associated with a given cow on the same scale as estimated survivorship.

**FIGURE 4:**
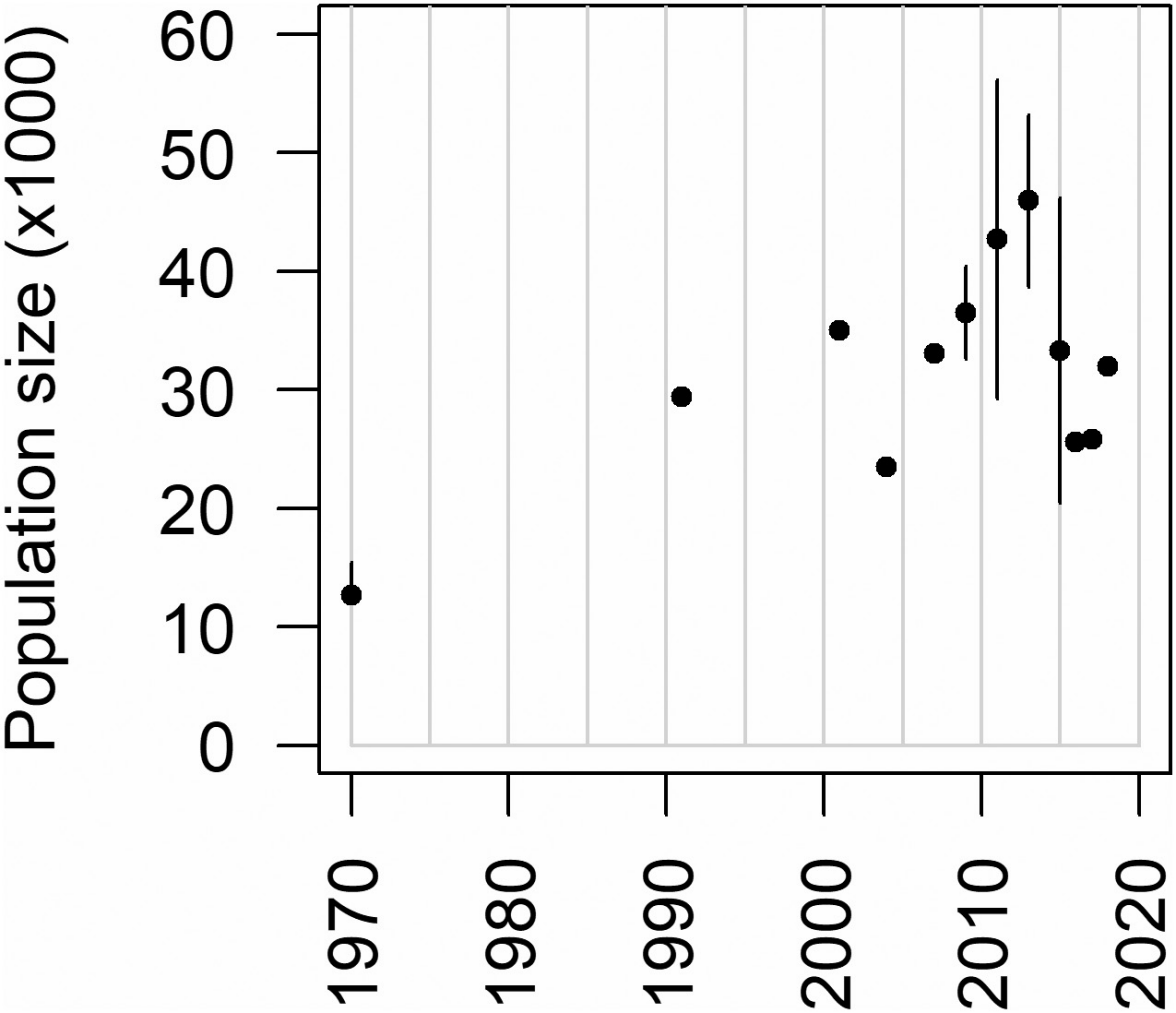
Population size estimated from aerial surveys between 1970 and 2018. The single error bar in 1970 represents Berry's qualitative speculation as to how much the true population size may have been above their actual count. Error bars for the surveys carried out between 2009 and 2015 represent Viljoen's 95% confidence interval based on the Jolly's II method for sample counts. Surveys from 2016 to 2019 were total counts and hence did not quantify confidence intervals.

**FIGURE 5:**
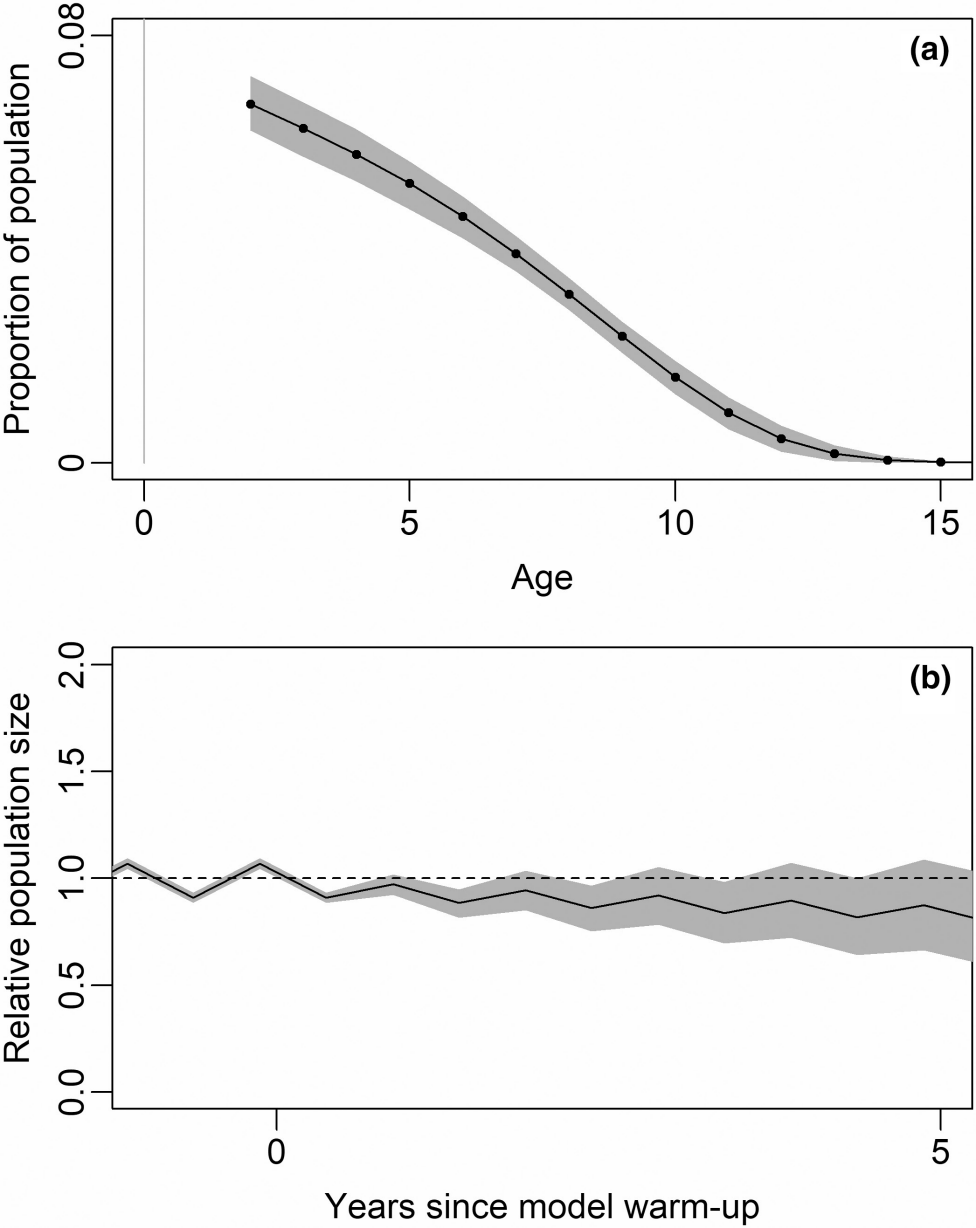
Demographic population model predictions derived from estimated vital rates: (a) stable age structure after model warm‐up, (b) population trajectory, with abundance represented as “relative population size” normalized to a value of 1 before each iteration of the warm‐up period, and thereafter allowed to vary. The 50‐year warm‐up period achieved stabilization of the age distribution to provide a consistent starting point for estimation of population growth rate.

**FIGURE 6:**
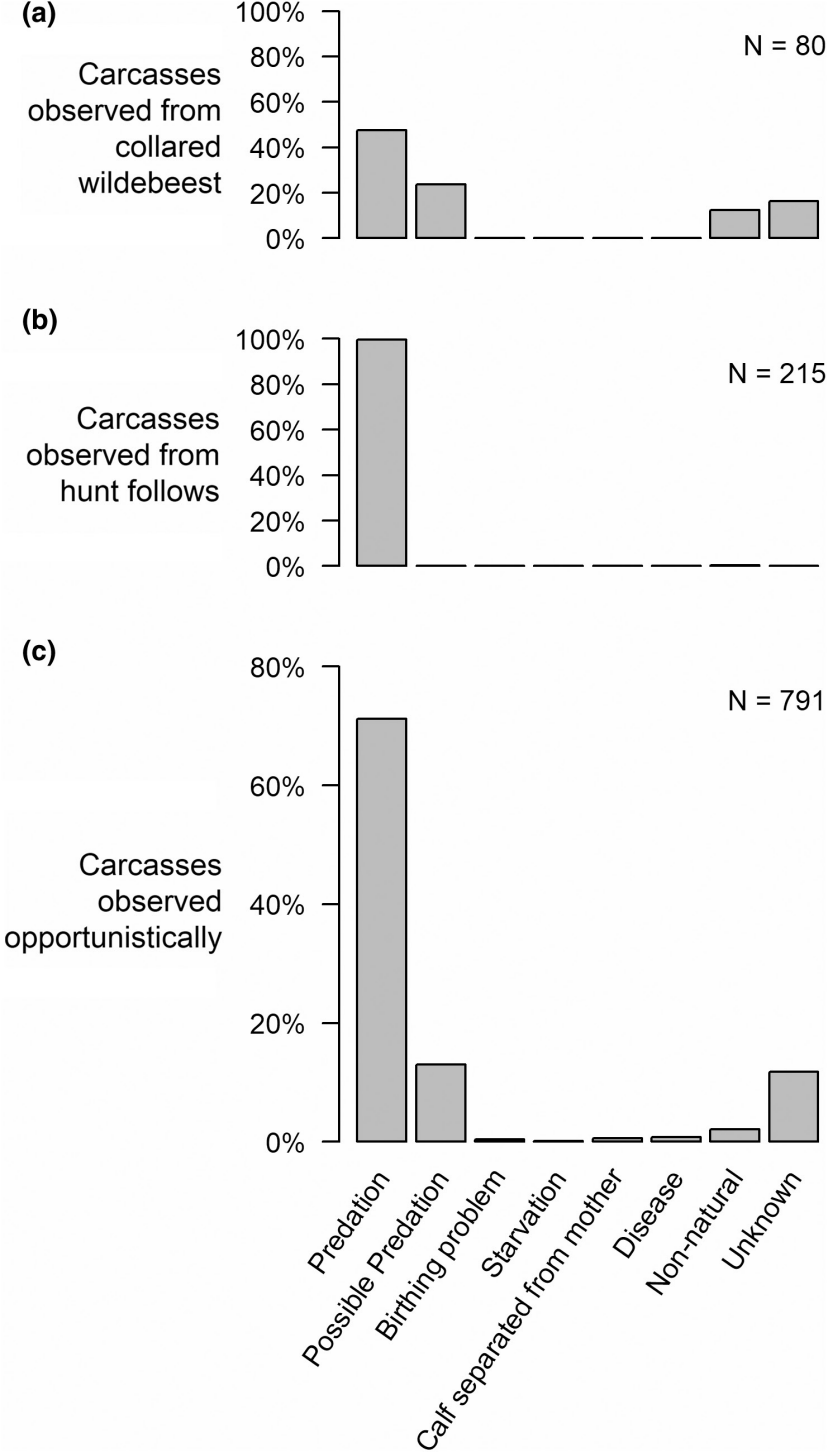
Estimated cause of death for 1086 documented wildebeest mortalities discovered by various means. “Birthing problems” included stillborn calves and mothers who died giving birth. “Calf starvation” includes calves that died after losing their mothers. Causes listed as “non‐natural” include poaching, harvest for lion management purposes, entanglement in a fence, and encounter with a temporary electric fence used around a temporary airstrip in 2011–2012.

Wildebeest movements and migrations (Figure 1) were consistent with prior descriptions (Droge et al., 2019; M'soka et al., 2016). Wildebeest calving typically occurred in the northern‐central portions of the park, with herds arriving on their wet season range several weeks after peak calving. The peak of the calving season was approximately October 1st.

### 3.1 Adult survival estimated from collared cows

Adult annual survival declined steadily from 91% (89%–92%, Model CC) at age 2 years to 57% (51%–59%) at age 10 years thereafter dropping more sharply to 2% (0%–13%) at age 16 (Figure 2). Mean cow age at mortality was 8.6 years. There was substantial evidence that hazard to adults was 27% higher in the wet season (LER [*β*_S_ > 0] = 0.70, Model CC.S). There was strong evidence that hazard to adults was 45% higher in the southeastern portion of the wildebeest range (LER [*β*_L_ < 0] = 1.44, Model CC.L). Interannual variability was pronounced—the mean ratio in hazard between any two years was 1.53 (LCL_0_ = 1.30, Model CC.Y) and the ratio in hazard between the highest and lowest years was 2.77 (LCL_0_ = 1.85, Model CC.Y).

### 3.2 Fecundity, calf survival, and recruitment based on herd‐composition data

From herd composition data, mean fecundity was estimated to be 0.68 (0.57–0.82), mean survivorship to age 1.0 was estimated to be 0.56 (0.47–0.65), mean recruitment to age 1.0 was estimated to be 0.38 (0.36–0.40), and mean recruitment to age 2.0 was estimated to be 0.31 (0.27–0.36) (Model HC). There was strong evidence for pronounced interannual variation in each of these rates, that is, that mean absolute between‐year differences in fecundity, survivorship, and recruitment were at least 0.11, 0.11, and 0.08, respectively (LCL_0_, Model HC.Y). Estimated rates for specific years are listed in Table A2. The degree of interannual variation was similar between calves and adults; the mean ratio in first‐year calf hazard between any two calendar years was 1.64 (LCL_0_ = 1.41, Model CC.Y), which is similar to the value of 1.53 reported for adults (above).

### 3.3 Fecundity, calf survival, and recruitment based on calf‐detection data

From calf‐detection data, mean fecundity, survivorship, and recruitment were 0.68 (0.61–0.74), 75, 0.59 (0.51–0.68), and 0.40 (0.34–0.46), respectively (Model CD). These estimates very closely match those obtained from herd‐composition data. There was strong evidence for pronounced interannual variation in each of these rates. Mean between‐year differences were at least 0.18, 0.14, and 0.16 for fecundity, survivorship, and recruitment, respectively (LCL0, Model CD.Y).

There was decisive evidence for calf dissociation from their mothers in the last quarter of the year (LER > 2, Model CD.Y). Most of the calves were estimated to have dissociated from their mothers well before the following calf season (Figure 3) (Note that this does not bias estimation of recruitment, because the model estimates dissociation separately from recruitment). The estimated probability, *p*, of correctly detecting a cow's calf was 0.89 (0.88–0.90) and the estimated probability, *q*, of falsely detecting another cow's calf was approximately 0.02 (0.02–0.03). There was more uncertainty in vital rates estimated directly from the calves of collared cows as compared with rates estimated from herd counts because the sample size was much smaller for direct observation of collared cows.

There was no evidence for a decline in fecundity with cow age. Mean fecundity before and after age 8 was 0.67 and 0.72, respectively (based on sample sizes of 189 and 60, respectively).

### 3.4 Longevity and life expectancy

The estimated age exceeded by only 0.1% of females was 14.41 years (CI 13.27–15.47, Figure 5a). The estimated life expectancy at birth was 3.71 years (CI 2.43–4.81).

### 3.5 Population trend, and verification of self‐consistency of estimated vital rates

Aerial population survey data revealed no clear evidence of a long‐term upward or downward trend in the 27 years between 1991 and 2018 (Figure 4). Although detectability bias can be considered negligible due to the open nature of the grassland habitat of the area, observer bias (either through overcounting or undercounting) may have played a role in the wide fluctuations in population estimates, at least until photogrammetry was initiated from 2016. Given the very wide confidence intervals around the sample counts conducted between 2009 and 2015 (Figure 4), it is more likely that the clumped distributions of very large herds of wildebeest did not meet the assumptions of the SRF sampling technique employed. Nevertheless, the vital rates estimated from herd counts and collared cows were found to be self‐consistent, in that they led to a predicted population trajectory that was not clearly crashing or booming in a manner that would be inconsistent with the aerial survey data (Figure 5b). The expected value of the annual population growth rate was 0.976 with a credible interval (0.930–1.024) that spanned 1.000 (Figure 5b). Population turnover was approximately 0.153 (0.137–0.167), estimated as the net annual mortality from model runs with an approximately stable population growth rate (0.98–1.02), and comparable with estimated annual recruitment of 0.156 females per adult female.

### 3.6 Causes of death

We examined cause of death for 1086 carcasses (Figure 6). This included 981 animals with a known, natural cause of death, that is, where the cause of death could be reasonably ascertained and was not suspected of being unnatural (e.g., poaching). Of these, 67 were from collared cows, 215 were from hunt follows, and 699 were discovered opportunistically. The data from collared cows and opportunistically encountered carcasses in particular are relatively unbiased indicators of the causes of mortality in the population. Known natural collared cow cause of death was 66.7% predation and 33.3% possible predation. Opportunistically encountered carcass cause of death was 82.7% predation and 15.1% possible predation, for a total of 97.8% predation or possible predation. There is thus a strong indication that most wildebeest in the system die from predation. Starvation only accounted for 0.8% of all unbiased known natural causes of death.

## 4 DISCUSSION

Despite being Africa's most abundant migratory herbivore and an iconic species for much of the continent's premier wildlife areas, no long‐term intensive individual‐based demographic studies—as per the recommendations of Gaillard (2013)—have been conducted on blue wildebeest to our knowledge. The GLE supports one of the largest remaining wildebeest migrations, but prior to this study the demography of the population and factors limiting its recovery were almost completely undescribed. From this baseline we conducted intensive studies of collared cows and their calves, concurrent with collection of herd size and composition data and comparisons with aerial surveys to estimate fecundity, recruitment, and survival and to evaluate factors limiting population recovery. In concert, this work provided some of the first detailed demography of the species and demonstrated that the Liuwa wildebeest population had strong age‐structured rates of survival and variable rates of calf recruitment. While we have not yet specifically identified factors regulating inter‐annual population dynamics (sensu Sinclair et al., 2006), we have established that the GLE wildebeest population exhibited pronounced geographic and seasonal patterns of survival and was strongly limited by predation and predation risk effects.

Large, long‐lived mammals typically have relatively constant rates of adult survival and variable rates of annual juvenile survival and recruitment (Eberhardt, 2002); however, few aging techniques have been applied to African ungulates to allow precise age estimation of adults, and a novel age estimation technique from tooth allometry enabled evaluation of age‐structured vital rates for wildebeest (Christianson et al., 2018). The estimated age exceeded by only 0.1% of females (14.4 years, CI 13.3–15.5) was somewhat consistent with the ages we obtained from tooth cementum annuli (oldest tooth aged: 11.1 years) and one to two years shorter than maximum longevity values reported from other populations: 17 years (Talbot & Talbot, 1963), 16 years (Watson, 1967, as cited by Estes, 2014), 16 years (Andere, 1975), 16–17 years (Attwell, 1982). The estimated life expectancy at birth (3.7 years, CI 2.4 to 4.8) was shorter than other values in literature: 5.4 years (Andere, 1975), 7 years (Watson, 1967 as cited by Estes, 2014). Not surprisingly, wildebeest cows exhibited strong age‐based survival rates, with older cows having lower rates of survival (Figure 2). As with prior studies in the GLE, predation was strongly age‐class specific, with hyenas and lions predating primarily adult wildebeest, and cheetah and wild dogs primarily calves and yearlings (Creel et al., 2017, 2019; Droge et al., 2017a). The one exception to this was in the first few weeks of calving, when hyenas focused heavily on vulnerable newborn calves before resuming typical patterns of larger adult age classes; this was consistent with findings from the Serengeti‐Mara wildebeest studies (Estes, 2014). Numerous studies have demonstrated age‐based predation on adult ungulates, with coursing predators typically taking older animals in the adult age class in addition to the juvenile age class (Becker et al., 2009; Husseman et al., 2003; Kruuk, 1972; Kunkel et al., 1999; Schaller, 1972). Given spotted hyena (a coursing predator) were the dominant predator on wildebeest this would be expected, and the observed age dependency of mortality risk supported this (Figure 2b). While it was difficult to determine cause of death for calves of collared cows, concurrent lines of investigation indicated predation was again the main cause of mortality. Spatial data on wildebeest indicated that calving typically occurred in the northern portion of the LPNP, where predation risk was considerably lower, and calf: cow ratios quickly dropped in the first few weeks before slowing. Predation was most intense in the first few weeks following calving before hyena also resumed hunting primarily larger age classes (ZCP *unpublished data*). This supports identical findings from Estes (2014) indicating that hyena specialized on calves during the short window of the first few weeks of life, before predating larger age classes predominantly.

Bolger et al. (2008) emphasized the need for evaluating factors limiting migratory ungulates across all phases of migration, given that these factors can vary with different phases, and our results support this recommendation. Adult wildebeest survival varied substantially between the wet and dry season range, primarily due to the significant increase in predation during the wet season. Herds spent the wet season in a relatively small area comprising the ecosystem's highest density of predators, before migrating north with the onset of the dry season into a much lower predator density. Thus, migration to the more northern areas during the dry season and for calving is likely undertaken at least in part to escape the intense predation pressure of the wet season range. The determination of whether predators limit prey rests on several observations (Mills, 2007): the predation rate must be high, a substantial amount of the predation must be additive and not compensatory, and the killed prey must have high reproductive value. The evidence strongly supports each of these criteria. The predation rate is high; annual population mortality is high (estimated at 15.3%) and most of this is due to predation (81.4% or 98.0%, depending on how “possible” predations are accounted). If the predation was compensatory, we would expect evidence of starvation in the population, but starvation only accounted for 0.8% of known natural mortalities observed by unbiased means. Finally, high reproductive value individuals are predated, with the average age at mortality of predated collared females being 7.80 years and no evidence for a decline in fecundity beyond this age. Additionally, while direct predation is the most obvious impact of predators, significant predation risk effects were present (Creel et al., 2017, 2019; Droge et al., 2017a, 2017b, 2019; M'soka et al., 2017) and even if resource limitation was detected, this could be as the result of wildebeest selecting areas of poorer habitat quality to avoid predictable areas of high predation risk, consistent with the Control of Risk Hypothesis (Creel, 2018).

While predation and predation risk effects exerted a strong effect on wildebeest demography and likely migration, we cannot rule out the importance of other drivers in seasonal movements. Ecological factors such as access to forage and its component variables such as precipitation, fire, surface water availability, and nutrients have been identified as key drivers for migration for wildebeest and other migratory herbivores (Geremia et al., 2014; Holdo et al., 2009a, 2009b; Hopcraft et al., 2015; Sinclair & Norton Griffiths, 1982). It is possible that the indigestible content of wet season forage (including water content) was so high that animals could not easily maintain nutrition (Hopcraft et al., 2001, 2010). Given the extensive flooding of the GLE in the rains—and subsequent high water‐content of forage—it is possible that nutrient limitation drove wildebeest herds to occupy areas with high predation risk in order to fulfill energy, protein, and possibly mineral requirements. While it is not the focus of this paper, future analyses will be focused on the drivers of wildebeest spatial dynamics and migration in the GLE.

Poaching of wildebeest also impacted wildebeest survival in the GLE and was conducted with a variety of methods ranging from firearms (primarily shotguns) to wire‐snares set around waterholes and woodlands, and spears and dogs. Depending on the method of poaching, the impacts on wildebeest populations are likely to vary, with snares being largely nonselective and firearms likely being more focused on larger adult wildebeest. With both methods significant numbers of wounded animals can occur, complicating evaluations if they are subsequently predated by opportunistic predators like hyenas. Thus, while predation was the dominant cause of mortality, the impact of poaching should not be discounted and needs continued attention. This is particularly so because of the rapid rates of human‐induced ecological change occurring throughout Zambia (Watson et al., 2015, and similar unpublished subsequent data for western and northwestern Zambia) and a strong association between these changes and consequent poaching pressure on wildlife populations (Watson et al., 2013).

The absence of wildly unrealistic modeled population trajectories constitutes support for the validity of the vital rate estimates and their associated posterior distributions. A stable population growth rate (*λ* = 1.0) was included within credible interval (0.930–1.024) for the population growth rate implied by the vital rate estimates and under the expected value of 0.976 it would take 10 years for the population to decline more than 20%. This is by no means assured, given the disparate investigations underlying these estimates—including tooth allometry, herd counts, and traditional mark‐resight survival estimation—and the absence of any stabilizing density‐dependence mechanism built into the model. It also supports the notion apparent from the aerial population survey data that there is no clear evidence of upward or downward trend in GLE population size at present, notwithstanding the considerable imprecision brought about by large sample errors in SRF sampling and potential for observer bias through over‐ or underestimation (particularly of very large herds of animals) inherent in aerial surveys prior to the use of photogrammetry (Lamprey et al., 2020; Schuette et al., 2018). A more‐detailed future analysis could consider both non‐zero yearling fecundity, and 2‐year‐old fecundity being lower than that of older adults—both of which have been noted in the literature with substantial variability between years and between systems (Mduma et al., 1999; Mills & Shenk, 1992; Tambling & Du Toit, 2005), and for which we have a limited number of supporting observations from the GLE.

Nevertheless, the increased resource protection efforts for both the GLE and its wildlife since 2003 has resulted in very high rates of survival for wildebeest‐dependent carnivores such as hyena (M'soka et al., 2016), and no evidence of population declines, indicating carnivore populations are likely tracking herbivore populations that are stable to increasing. The influence of predation on wildebeest demography relative to resource‐limitation was in striking contrast to studies elsewhere such as that of Mduma et al. (1999) which found forage to be the primary limiting factor for Serengeti wildebeest. Whether the strong predation limitation is a factor of current wildebeest population size or transitional recovery dynamics, or whether it reflects differences in ecological dynamics in the GLE versus other populations remains to be seen. Similarly, dynamics of wildebeest could change significantly with changes in diet and preference of hyenas and other large carnivores. Increases in predation on other relatively abundant species such as zebra could either exert a stabilizing influence on wildebeest through prey switching or increase predation impacts through apparent competition. Continued restoration of both the migrant and resident herbivore community in the GLE will likely have an array of impacts on predator–prey dynamics (Creel et al., 2019).

Large herbivore migrations are in decline worldwide and while Africa has the largest number of remaining migrations, only one—the Serengeti‐Mara Ecosystem—is mostly protected (Harris et al., 2009), although even this range is at risk from road developments and other human impacts (Holdo et al., 2011; Hopcraft et al., 2015). Harris et al. (2009) identified key actions needed to conserve migratory populations, specifically the need to secure seasonal range, resource protection, governmental support, and minimizing fences. Migratory populations are typically more abundant than resident ones as they can likely avoid the carrying‐capacity restraints imposed on residents by tracking resources and avoiding predation; however, when constraints or barriers to migration occur, dramatic declines and even collapses in populations can occur (Bekenov et al., 1998; Berger, 2004; Berry, 1997; East, 1988; Estes & East, 2009; Fryxell & Sinclair, 1988; Whyte & Joubert, 1988), and wildebeest migrations appear particularly sensitive to disruption (Holdo et al., 2011; Morrison et al., 2016; Morrison & Bolger, 2014; Thirgood et al., 2004). The GLE wildebeest population is strongly limited by predation, particularly on their wet season range that encompasses the system's highest densities of predators. Rapid human encroachment in the form of agricultural conversion in the UWZGMA that comprises much of the GLE wildebeest dry season range and lies completely outside the LPNP is threatening both the current migratory range of the wildebeest and any potential expansion and resumption of historical transfrontier migrations. If wildebeest were to be decoupled from their migratory range outside the LPNP then the population would likely be subjected to intense year‐round predation from a growing carnivore population in the south, which would likely drive declines if herds were not able to escape high levels of predation on their dry season range and during calving. Consequently, there is an urgent need for protection of the entire GLE wildebeest range in order to secure the long‐term viability of this keystone species in a rapidly changing ecosystem. Increased protection of current and potential dry season range outside LPNP should be considered the highest priority for conservation of wildebeest and other dependent species (Becker et al., 2017).

## AUTHOR CONTRIBUTIONS

**Fred Watson:** Conceptualization (lead); data curation (equal); formal analysis (lead); funding acquisition (supporting); investigation (equal); methodology (equal); software (lead); visualization (lead); writing – original draft (lead); writing – review and editing (equal). **Matthew S. Becker:** Conceptualization (lead); data curation (supporting); formal analysis (lead); funding acquisition (lead); investigation (equal); methodology (equal); project administration (lead); resources (lead); supervision (lead); visualization (supporting); writing – original draft (lead); writing – review and editing (equal). **Daan Smit:** Data curation (equal); investigation (equal); methodology (supporting); writing – review and editing (equal). **Egil Droge:** Data curation (equal); investigation (equal); methodology (supporting); writing – review and editing (equal). **Teddy Mukula:** Data curation (supporting); investigation (supporting); writing – review and editing (supporting). **Sandra Martens:** Investigation (supporting); writing – review and editing (supporting). **Shadrach Mwaba:** Data curation (supporting); investigation (supporting); writing – review and editing (supporting). **David Christianson:** Conceptualization (supporting); writing – review and editing (supporting). **Scott Creel:** Conceptualization (supporting); funding acquisition (supporting); writing – review and editing (supporting). **Angela Brennan:** Data curation (supporting); funding acquisition (supporting); investigation (supporting); methodology (supporting); writing – review and editing (supporting). **Jassiel L J M'soka:** Investigation (supporting); methodology (supporting); writing – review and editing (supporting). **Angela Gaylard:** Writing – review and editing (supporting). **Chuma Simukonda:** Conceptualization (supporting); supervision (supporting); writing – review and editing (supporting). **Moses Nyirenda:** Conceptualization (supporting); funding acquisition (supporting); writing – review and editing (supporting). **Bridget Mayani:** Data curation (supporting); investigation (supporting); writing – review and editing (supporting).

## CONFLICT OF INTEREST

The authors declare that they have no competing interests in relation to the publication of this paper.

## Figures and Tables

**FIGURE A1:**
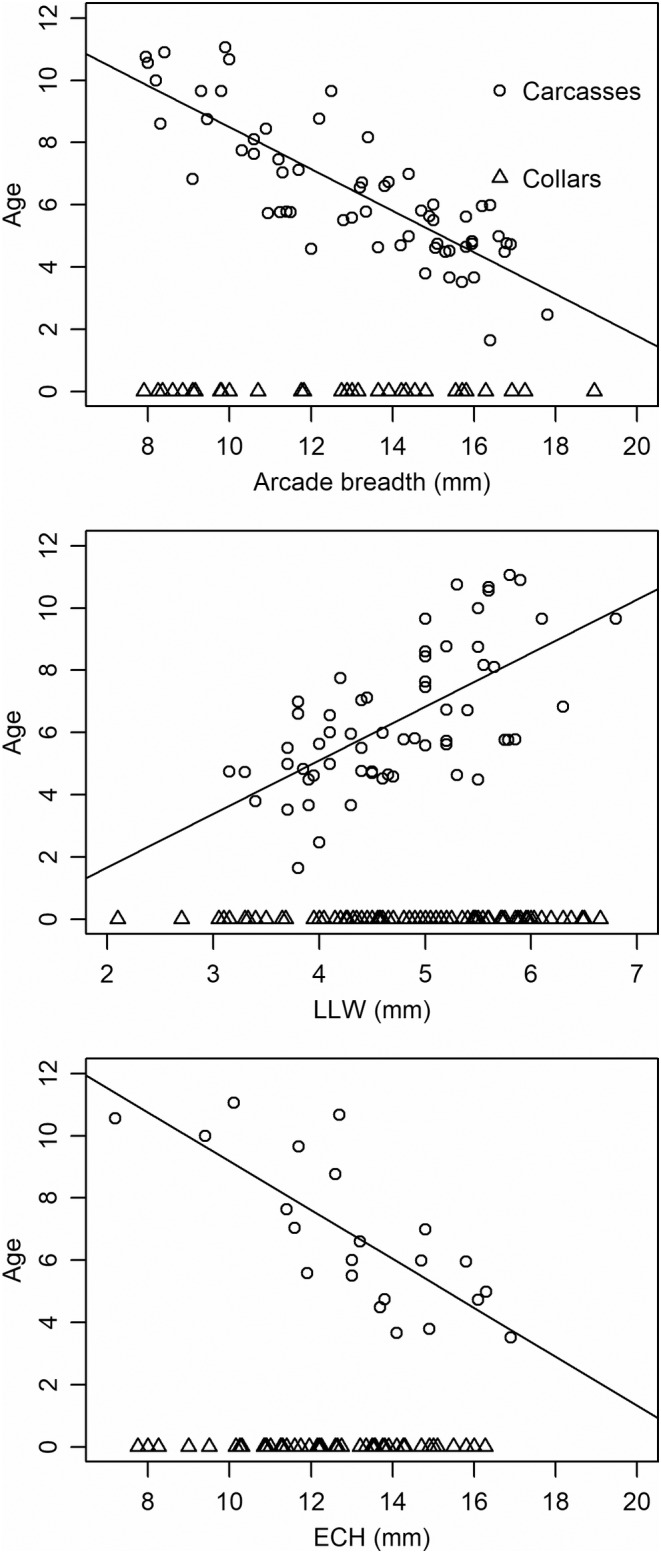
Estimation of wildebeest age (years) from the dimensions of teeth obtained from found carcasses. The dimensions for collared wildebeest at the time of collaring are also given, in order to show that age estimation for collared animals did not rely on excessive extrapolation beyond the range of measurements available from carcasses.

**FIGURE A2:**
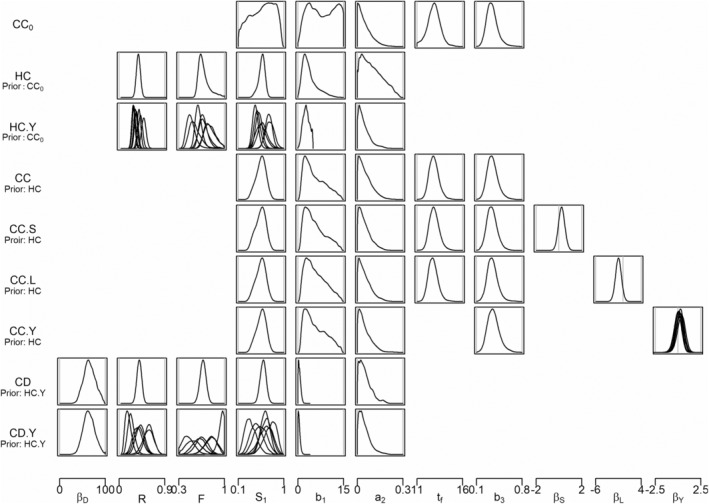
Posterior parameter distributions for a sequence of Bayesian models of wildebeest demography. Model names are indicated at left and parameter symbols are indicated at the bottom of the figure—See text and Table A1 for explanation of terms. Parameter estimation relied on a sequential Bayesian approach in order to estimate distributions of parameters that are determinant of both calf survival and adult survival and thus informed by multiple types of observation.

## Data Availability

Data supporting the results of this paper are archived at Dryad at https://doi.org/10.5061/dryad.0k6djhb3f.

## APPENDIX A

### Age of collared cows

We estimated the age of collared cows using tooth measurements in a two‐stage process based on the cementum annuli of the first and second incisors (Christianson et al., 2018). The first stage utilized teeth extracted from adult wildebeest carcasses detected in the course of fieldwork on predators and wildebeest. These teeth were aged at the Matson Lab (Manhattan, MT, USA) using cementum annuli techniques, from which we developed relationships between these age estimates and the physical dimensions of the teeth. Teeth from found carcasses ranged in age from 1.1 to 11.1 years, based directly on cementum annuli measurement. The second stage utilized tooth measurements taken from immobilized wildebeest during collaring and applied the carcass‐based relationships to yield an estimated age for each collared wildebeest. We made slight adjustments to the lab‐reported ages to accommodate the constraint that all wildebeest in the study system are born at a certain time of year (October). We measured three tooth dimensions: arcade breadth (AB), labio‐lingual width (LLW), and emergent crown height (ECH) (Christianson et al., 2018). Each of these can be measured both with a carcass tooth in hand, and from the in‐situ teeth of an immobilized animal. We regressed cementum age against each of the three dimensions in turn (Figure A1) and found that AB explained the most variation (*R* ^2^ = 0.68, *N* = 61, *p* < .0001), followed by ECH (*R* ^2^ = 0.59, *N* = 61, *p* < .0001) then LLW (*R* ^2^ = 0.41, *N* = 61, *p* < .0001). We, therefore, used AB as the primary basis for age estimation for collared animals, except where there were data gaps in AB, in which case we used ECH, and then LLW, as necessary. A reviewer suggested combining all three metrics in order to achieve a more accurate estimate. This would be worth exploring more, although we note that certain multimetric approaches would be precluded by data gaps. Some animals were recollared after a few years, in which case we used the age estimate from the first collaring because these were generally more precise. Age estimates from recollaring generally agreed with those from initial collaring but in some cases were relative underestimates. Finally, we qualitatively compared the distribution of tooth dimensions between carcasses and collared animals to verify that the carcass‐derived teeth were representative and that age‐estimation for collared animals would not rely on excessive extrapolation.

### Model for survival and recruitment

We quantified wildebeest survival by fitting a single survival model to three different types of field measurements, as described below. The model was of the form introduced by Siler (1979), whereby total hazard, *h*, is the summation of terms for (1) immaturity hazard, (2) constant hazard, and (3) senescence hazard:

$$h=a_{1}\exp\left(-b_{1}t\right)+a_{2}+a_{3}\exp\left(-b_{3}t\right)$$

where *t* represents age in years, and *a*_1_, *b*_1_, *a*_2_, *a*_3_, *b*_3_ are parameters with subscripts representing the three forms of hazards listed above. We use the terms “hazard” and “mortality risk” interchangeably depending on context, the former being more prominent in mathematical survival analysis.

The hazard function leads to a function for survivorship, *S* (Lee & Wang, 2003):

$$\begin{array}{c} S=\exp\left(-\int_{x=0}^{t}h\;\mathrm{dx}\right)\\ \;=\exp\left(-\left(\frac{a_{1}}{b_{1}}\left(1-\exp\left(-b_{1}\;t\right)\right)+a_{2}\;t+\frac{-a_{3}}{b_{3}}\left(1-\exp\left(b_{3}\;t\right)\right)\right)\;\right) \end{array}$$

In the original Siler model, parameters *a*_1_, *a*_2_, and *a*_3_ specify the magnitude of each hazard, and parameters *b*_1_ and *b*_3_ specify the rate of reduction in immaturity hazard and increase in senescence hazard, respectively. We expressed *a*_1_ and *a*_3_ as a function of more biologically meaningful parameters *S*_1_ and *t*_*f*_, where *S*_1_ specified the first‐year survivorship, and *t*_*f*_ specified the time until survivorship fell to a specified low level, *f* = 0.01 (Barlow & Boveng, 1991; Becker et al., 2013). Given *S*_1_ and *t*_*f*_, as well as *b*_1_, *a*_2_, and *b*_3_, values of *a*_1_ and *a*_3_ can be calculated according to re‐arrangement of the original survivorship function:

$$a_{1}=\frac{b_{1}}{1-\exp\left(-b_{1}\right)}\left(-\log\left(S_{1}\right)-a_{2}-\frac{a_{3}}{b_{3}}\left(1-\exp\left(b_{3}\right)\right)\right)$$

$$a_{3}=\frac{-b_{3}}{1-\exp\left(-b_{3}t_{f}\right)}\left(-\log\left(f\right)-a_{2}t_{f}-\frac{a_{1}}{b_{1}}\left(1-\exp\left(b_{1}t_{f}\right)\right)\right)$$

These two equations are interdependent, but their solution converges in two iterations under typical values of the known parameters.

We explored models with several subsets and variants of the survivorship function (Table A1). Subsets excluded one or more of the three primary hazards. Variants modulated the Siler hazard with additional influences due to season, year, location, and dissociation of calves from their mothers. A season effect, *X*_*S*_ ∈{0,1}, was dichotomized into dry and wet seasons according to a parameter *β*_*S*_:

$$h_{S}=h\times \exp\left(\beta_{S}\left(X_{S}-0.5\right)\right)$$

A year effect, *X*_*Y*_ ∈{2012, 2013, …}, was represented with a vector of fitted parameters ***β***_*Y*_, and a fixed reference longevity, *t*_*f*,ref_, arbitrarily set at 13.3 years so as to center the ***β***_*Y*_ values approximately about zero:

$$h_{Y}=h\times \exp\left\{\begin{array}{cc} \beta_{Y,2012} & X_{Y}=2012\\ \beta_{Y,2013} & X_{Y}=2013\\ \ldots & X_{Y}=\ldots \end{array}\right.$$

To explore spatial variation in hazard, a location effect, *X*_*L*_, was represented with a fixed parameter *X*_*L,ref*_ denoting a reference location, and a fitted parameter *β*_*L*_:

$$h_{L}=h\times \exp\left(\beta_{L}\left(X_{L}-X_{L,\mathrm{ref}}\right)\right)$$

Location was represented as the relative distance, *X*_*L*_ ∈ [0,1], along the major axis of wildebeest migration measured from an arbitrary point at the southeast limits of the migration. The reference location was chosen as the median relative distance, *X*_*Lref*_ = 0.355.

All secondary effects were centered around zero and then exponentiated to prevent negative hazards as indicated in the above equations.

A calf dissociation effect was represented as a “hazard.” Calves begin to dissociate from their mothers at approximately 0.75 years of age (Estes, 2014). This has the same numerical effect as mortality in observations where calf association with individual cows is used as an individual metric of fecundity and survivorship. So, we modeled it as such, using a fixed parameter, *t*_*D*_ = 0.75 years, for the time of commencement of dissociation and a fitted parameter, *β*_*D*_, for the rate of dissociation:

$$h_{D}=h+\beta_{D}\max\left(0,t-t_{D}\right)$$

with survivorship (under *a*_2_ = 0 and *a*_3_ = 0):

$$S=\exp\left(-\left(\frac{a_{1}}{b_{1}}\left(1-\exp\left(-b_{1}\;t\right)\right)+\frac{\beta_{D}}{2}{\left(\max\left(0,t-t_{D}\right)\right)}^{2}\right)\;\right)$$

To model recruitment, *R*, we incorporated a fecundity parameter, *F*, that was either a scalar or a vector of separate fecundity values for each year, depending on the model variant:

R=F×S

### Model fitting to known fates of adults

We estimated adult survival using a known‐fates approach, assuming perfect detection of living animals and their deaths. We assured perfect detection through frequent attempts to relocate each collared animal, rotating through all animals and focusing on the ones seen least recently, and grouping the resulting relocation data into six‐month periods. Six months is longer than the time it took to determine the state of all collared animals, given the level of year‐round search effort we maintained (800–1100 person‐days per year). We assumed a closed population. No other populations are nearby and opportunity for emigration is strongly limited by landscape features. Four collars were lost due to either malfunction or destruction by predators, scavengers, or poachers. In these cases, we removed the animal from the study at the start of the period in which the collar loss occurred. Thus, the animal had a known fate (survival) through to the period prior to the collar loss. We encountered no evidence that collar loss was correlated with fate or predictor variables such as season, location, or age.

The likelihood function for observation of survivorship over a known period of time to a known fate (living, or died at a known time) is Rodriguez (2010):

$$L_{i}\left(\Theta |t,Z\right)=\prod_{j=1}^{N}S_{i,j}\;{h_{i,j}}^{Z_{i,j}}$$

$$S_{i,j}=\exp\left(-h_{i,j}\;t_{i,j}\right)$$

$$Z_{i,j}=\left\{\begin{array}{cc} 1 & \text{Wildebeest}\;i\;\text{died in Semester}\;j\\ 0 & \text{Otherwise} \end{array}\right.$$

where *t*_*i*,*j*_ is the amount of time (years) in which wildebeest *I* was in the study during semester *j*. This value is 0.5 years for wildebeest who were collared before a semester and survived past the end of that semester, but <0.5 years for wildebeest either collared or determined to have died during a semester (or both), and 0 in semesters before collaring or after death.

### Model fitting to observations of herd composition

We estimated recruitment from fecundity and calf survival in two ways: (1) from calf: cow ratios in herd counts, and (2) from intensive monitoring of the calves of collared cows, thereby providing two concurrent and independent measures (Garrott et al., 2009). Observed calf: cow ratios are an indicator of the progression of recruitment from birth toward maturity. Wildebeest are synchronous breeders and in the GLE all calves are born within a few weeks of the first of October, so the calf: cow ratio around this time is an indicator of fecundity, and the subsequent decline in this ratio is an indicator of calf survivorship (Some temporal extrapolation is involved in the estimation, because of the exclusion of herd count data from the aforementioned September 1 to October 30 period near calving season when detecting and aging of calves is less reliable).

Animals classified as calves or cows in herd counts can be viewed as an outcome of a binomial process with a probability (*θ*) that each calf or cow (*Y*) in the herd count will be either a calf (*Y* = 1) or a cow (*Y* = 0):

$$Y∼\text{Bernoulli}\left(\theta \right)$$

$$\theta =R/\left(1+R\right)$$

A standard binomial likelihood accompanies such a model:

$$L\left(\Theta |Y\right)=\theta^{y}{\left(1-\theta \right)}^{n-y}$$

where Θ is a vector of all model parameters, *y* is the number of calves, and *n* is the number of calves and cows. These likelihoods were raised to a power of a 0.2 to compensate for the estimated rate of repeat records of the same animal in a given year. Recruitment was not a directly fitted model parameter. Rather, it was a model output computed from other modeled quantities depending on the fitting context—either from F and S under the calf detection data, or from *θ* under herd composition data (see below). Recruitment to specific ages 1 and 2 was denoted *R*_1_ and *R*_2_, respectively.

### Model fitting to detections of the calves of collared cows

The second way in which we estimated recruitment from fecundity and calf survival was to monitor the association of calves with individual cows, that is, by recording at each resighting of a collared cow the detection or nondetection of a calf associated with that cow. Calves stay with their mother for approximately 9 months, at which time they can begin to dissociate from their mother, until all are dissociated once new calves arrive. Twins and adoption do not occur (Estes, 2014, ZCP *unpublished data*) and we assumed that calves died if their mother died. With a few minutes of observation, it is generally straightforward to determine if a cow has a calf and to which cow a given calf belongs; we referred to this as a “true detection”. Such determinations are not infallible, however, and it possible to erroneously record another cow's calf as being associated with a specific collared cow. We referred to this as a “false detection”. We accounted for this with an observational model that incorporated true and false detection probabilities.

The collared‐cow calf detection model is not unlike traditional capture‐resight models, but the possibility of false detections necessitated special treatment via a likelihood function that accounted for the specific observational circumstances of our study using standard binomial and multinomial likelihood principles:

$$L\left(\Theta |Y,A\right)=\prod_{i=1}^{N}F\;L_{F,i}\left(\Theta |Y,A\right)+\left(1-F\right)\;L_{B,i}\left(q|Y\right)$$

where *F* is fecundity, *q* is the false detection probability, **Y** is a ragged detection matrix for *n*_*i*_ calf detections ($Y_{i,j}\in \left\{1,0\right\}$) in each of *N* calf‐years (*i*), and **A** is an identically sized matrix of the ages (in years) of calves at the detection occasions represented by **Y**. The overall likelihood (*L*) is a binomial combination of separate likelihoods (*L*_*F*_ and *L*_*B*_) representing the possibility that a cow in a given calf‐year was fecund or barren, respectively. In turn, the fecund likelihood considers all possible and detectable life histories (Z) for calf *i* observable on the occasions represented by **Y**:

$$\begin{array}{c} Z_{n_{i},z}=\left\{1_{1},1_{2},\cdots ,1_{z},0_{1},0_{2},\cdots ,0_{n_{i}-z}\right\},\text{for}\;z\in \left\{0,\cdots ,n_{i}\right\},\text{with the shorthand}\;Z=Z_{n_{i},z} \end{array}$$

where *z* indexes the last occasion when the calf was still alive. For example, the detectable life history for a calf that was born but never detected (*z* = 0) would be {0,0,…,0}; the history for a calf that died after the third sampling occasion (*z* = 3) would be {1,1,1,0,0,…,0}; and the history for a calf that survived at least through all sampling occasions (*z* = *n*_*i*_) would be {1,1,…,1}. A multinomial likelihood is summed over all of these possibilities, weighting each one by the appropriate interval of survivorship$,{\left(∆S\right)}_{i,z}$, between consecutive sampling occasions (Choquet et al., 2011; Fieberg & DelGuidice, 2011; Friedman, 1982):

$${\left(∆S\right)}_{i,z}={S^{*}}_{i,z+1}-{S^{*}}_{i,z+2}$$

$${S^{*}}_{i}=\left\{1,S_{i,1},S_{i,2},\cdots ,S_{i,n_{i}},0\right\}$$

$$S_{i,z}=\exp\left(-hA_{i,z}\right)$$

$$\sum_{z=0}^{n_{i}}{\left(∆S\right)}_{i,z}=1$$

which leads to an overall fecund likelihood:

$$L_{F,i}\left(\Theta |Y,A\right)=\sum_{z=0}^{n_{i}}\left({{\left(∆S\right)}^{i}}_{z}\;\prod_{j=1}^{n_{i}}\left(\begin{array}{c} \begin{array}{c} +\\ + \end{array}\\ \begin{array}{c} +\\ +\\ + \end{array} \end{array}\;\begin{array}{c} \begin{array}{c} \left(1-Z_{j}\right)\left(1-Y_{i,j}\right)\left(1-q\right)\\ \left(1-Z_{j}\right)Y_{i,j}q\\ Z_{j}\left(1-Y_{i,j}\right)\left(1-p\right)\left(1-q\right) \end{array}\\ \begin{array}{c} Z_{j}\;Y_{i,j}\;\left(1-p\right)q\\ Z_{j}\;Y_{i,j}\;p\;\left(1-q\right)\\ Z_{j}\;Y_{i,j}\;p\;q \end{array} \end{array}\right)\right)$$

where *p* is the true detection probability. The six individual summed terms in the fecund likelihood explore the six possible ways in which each detection ($Y_{i,j}\in \left\{1,0\right\}$), can arise from the calf being alive or not ($Z_{j}\in \left\{1,0\right\}$), the correct calf being detected (*p*), or another calf being detected (*q*) (given that the cow was fecund in that calf‐year). The barren fecundity is simpler, needing only to account for two detection possibilities, and no calf life history:

$$L_{B,i}\left(q|Y\right)=\prod_{j=1}^{n_{i}}\left(\left(1-Y_{i,j}\right)\left(1-q\right)+Y_{i,j}\;q\right)$$

At the outset, we considered that the two methods of estimating recruitment should lead to similar estimates, but they each may have different strengths. We considered herd counts to provide the best overall estimates of recruitment, being based on a very large sample size. Monitoring of the calves of individual cows is based on a much smaller sample size but is the only approach that allows incorporation of covariates that rely on movement history. It also provides a useful independent check on the herd‐count approach.

### Model fitting sequence

The model was fit to the different data sets using a sequential Bayesian approach, with the posterior parameter distributions from initial fits informing the prior parameter distributions of subsequent model fits (Daniels et al., 2014; Garrard et al., 2012; Kurota et al., 2009; Michielsens et al., 2008). The sequence was initiated by fitting the adult survival model to known‐fates data with noninformative priors and no inter‐annual variation (Model CC0, Table A1, Figure A2). This allowed some specification of the constant hazard (a2) parameter, which was found to be a necessary prior for convergence of the herd‐counts models (Models HC and HC.Y). Fitting of the HC model provided some specification of the first‐year survivorship (S1) which enable more precise fits of the known‐fates models (Models CC, CC.S, CC.L, and CC.Y) and estimation of associated adult survival parameters (βS, βL, βY). Finally, the calf‐detection models (CD and CD.Y) used posteriors from the HC.Y model as priors for the rate of reduction in immaturity hazard (b1) and constant hazard (a2) in order to achieve convergence, leaving the primary recruitment parameters (R, F, and S1) free to be estimated from noninformative priors. As an alternative to this sequential Bayesian approach, a single fit to an integral model with all data sets might be possible, but as Staton et al. (2017) demonstrated with a similar example in fisheries population dynamics, the results would likely be similar and the outward simplicity of an integrated approach might not outweigh the underlying computational expense. The sequential approach is computationally quicker and more assured of convergence because it explores a sequence of small low‐dimensional parameter spaces instead of one vast high‐dimensional parameter space. Our final sequence took about 7 h to run on a personal computer with a 2.8 GHz 4‐core processor, and model development involved hundreds of test runs.

**TABLE A1:** Summary of models fitted and parameters used.

Model name	Prior model	Age class	Observations used	Years of data	Number of parameters	Fecundity	Survivorship to age 1	Siler immature hazard	Siler immature rate	Dissociation age	Dissociation	True detect	False detect	Siler constant hazard	Longevity survivorship	Longevity time	Siler sensescnce hazard	Siler senescence rate	Season coefficient	Year coefficient	Reference longevity time	Location coefficient	Reference location
						*F*	*S*_1_	*a*_1_	*b*_1_	*t*_*D*_	*β*_*D*_	*p*	*q*	*a*_2_	*f*	*t*_*f*_	*a*_3_	*b*_3_	*β*_*S*_	*β*_*Y*_	*t*_*f,ref*_	*β*_*L*_	*X*_*L,ref*_
CC_0_	None	Adult	Collared cows	7	5	–	**1**	(1)	**1**	–	–	–	–	**1**	(1)	**1**	(1)	**1**	–	–	–	–	–
HC	CC_0_	Calf	Herd counts	6	4	**1**	**1***	(1)	**1***	–	–	–	–	**1***	–	–	–	–	–	–	–	–	–
HC.Y	CC_0_	Calf	Herd counts	6	13	**6**	**6**	(6)	**1**	–	–	–	–	–	–	–	–	–	–	–	–	–	–
CC	HC	Adult	Collared cows	7	5		**1***	(1)	**1***	–	–	–	–	**1***	(1)	**1**	(1)	**1**					
CC.S	HC	Adult	Collared cows	7	6	–	**1***	(1)	**1***	–	–	–	–	**1***	(1)	**1**	(1)	**1**	**1**				
CC.L	HC	Adult	Collared cows	7	6	–	**1***	(1)	**1***	–	–	–	–	**1***	(1)	**1**	(1)	**1**	–	–	–	**1**	(1)
CC.Y	HC	Adult	Collared cows	7	10	–	**1***	(1)	**1***	–	–	–	–	**1***	(1)	–	(1)	**1**	–	**6**	(1)	–	–
CD	HC.Y	Calf	Calf detection	6	7	**1**	**1**	(1)	**1***	(1)	**1**	**1**	**1**	**1***	–	–	–	–	–	–	–	–	–
CD.Y	HC.Y	Calf	Calf detection	6	17	**6**	**6**	(6)	**1***	(1)	**1**	**1**	**1**	**1***	–	–	–	–	–	–	–	–	–

*Note*: Numerals in the main body of the table indicate the number of parameters used. Boldface indicates fitted parameters. Parenthesis indicates derived parameters. Asterisks indicate parameters with priors informed by previous model run.

**TABLE A2:** Bayesian parameter estimates for several models of wildebeest demography.

Parameter	Year	Origin	Model	EV	LCL	UCL	LCL_0_	Pr > 0	LER	Model	EV	LCL	UCL	LCL_0_	Pr > 0	LER
*S*_1_		Fitted	CC	0.547	0.379	0.701										
*a*_1_		Derived	CC	3.030	0.420	6.237										
*b*_1_		Fitted	CC	5.512	0.884	10.998										
*a*_2_		Fitted	CC	0.055	0.000	0.118										
*t*_f_		Fitted	CC	12.862	12.006	13.736										
*a*_3_		Derived	CC	0.019	0.000	0.037										
*b*_*3*_		Fitted	CC	0.344	0.207	0.480										
*f*		Specified	CC	0.010	0.010	0.010										
*β*_S_		Fitted	CC.S	0.242	−0.189	0.666		0.833	0.698							
*β*_L_		Fitted	CC.L	−1.058	−2.041	−0.051		0.035	−1.444							
maxR(exp(*β*_Y_))		Derived	CC.Y	2.768			1.848									
meanR(exp(*β*_Y_))		Derived	CC.Y	1.528			1.298									
*h*_Y_/*h* = exp(*β*_Y_)	2013	Derived	CC.Y	1.559	0.607	2.514										
*h*_Y_/*h* = exp(*β*_Y_)	2014	Derived	CC.Y	1.382	0.560	2.191										
*h*_Y_/*h* = exp(*β*_Y_)	2015	Derived	CC.Y	1.263	0.513	1.998										
*h*_Y_/*h* = exp(*β*_Y_)	2016	Derived	CC.Y	1.037	0.448	1.607										
*h*_Y_/*h* = exp(*β*_Y_)	2017	Derived	CC.Y	1.136	0.522	1.753										
*h*_Y_/*h* = exp(*β*_Y_)	2018	Derived	CC.Y	1.029	0.423	1.627										
*h*_Y_/*h* = exp(*β*_Y_)	2019	Derived	CC.Y	1.321	0.636	1.997										
*F*		Fitted	HC	0.683	0.572	0.823				CD	0.675	0.611	0.737			
Range(*F*)		Derived	HC.Y	0.343			0.242			CD.Y	0.523	0.622		0.416		
MAD(*F*)		Derived	HC.Y	0.147			0.105			CD.Y	0.226	0.272		0.178		
*F*	2013	Fitted	HC.Y	0.549	0.419	0.683				CD.Y	0.451	0.308	0.577			
*F*	2014	Fitted	HC.Y	0.488	0.384	0.592				CD.Y	0.531	0.340	0.706			
*F*	2015	Fitted	HC.Y	0.606	0.532	0.686				CD.Y	0.654	0.500	0.801			
*F*	2016	Fitted	HC.Y	0.774	0.638	0.925				CD.Y	0.941	0.877	1.000			
*F*	2017	Fitted	HC.Y	0.783	0.646	0.917				CD.Y	0.796	0.647	0.959			
*F*	2018	Fitted	HC.Y	0.685	0.558	0.814				CD.Y	0.786	0.644	0.936			
*F*	2019	Fitted	HC.Y	0.678	0.576	0.777				CD.Y	0.577	0.421	0.735			
S1		Fitted	HC	0.562	0.465	0.645				CD	0.588	0.505	0.677			
Range(*S*_1_)		Derived	HC.Y	0.332			0.246			CD.Y	0.477			0.328		
MAD(*S*_1_)		Derived	HC.Y	0.146			0.109			CD.Y	0.201			0.14		
MR(*h*_1_)		Derived	HC.Y	1.635			1.406									
*S*_1_ = exp(−*h*_1_)	2013	Fitted	HC.Y	0.544	0.390	0.704				CD.Y	0.534	0.316	0.741			
*S*_1_ = exp(−*h*_1_)	2014	Fitted	HC.Y	0.583	0.420	0.741				CD.Y	0.343	0.161	0.518			
*S*_1_ = exp(−*h*_1_)	2015	Fitted	HC.Y	0.698	0.595	0.804				CD.Y	0.645	0.467	0.822			
*S*_1_ = exp(−*h*_1_)	2016	Fitted	HC.Y	0.511	0.410	0.603				CD.Y	0.630	0.488	0.778			
*S*_1_ = exp(−*h*_1_)	2017	Fitted	HC.Y	0.438	0.355	0.517				CD.Y	0.459	0.261	0.659			
*S*_1_ = exp(−*h*_1_)	2018	Fitted	HC.Y	0.475	0.374	0.574				CD.Y	0.645	0.427	0.860			
*S*_1_ = exp(−*h*_1_)	2019	Fitted	HC.Y	0.722	0.585	0.860				CD.Y	0.732	0.548	0.924			
R		Derived	HC	0.379	0.359	0.400				CD	0.396	0.337	0.457			
Range(*R*)		Derived	HC.Y	0.211			0.178			CD.Y	0.472			0.361		
MAD(*R*)		Derived	HC.Y	0.091			0.079			CD.Y	0.206			0.158		
*R*	2013	Derived	HC.Y	0.292	0.257	0.325				CD.Y	0.238	0.132	0.340			
*R*	2014	Derived	HC.Y	0.279	0.246	0.311				CD.Y	0.178	0.087	0.272			
*R*	2015	Derived	HC.Y	0.420	0.396	0.445				CD.Y	0.420	0.280	0.559			
*R*	2016	Derived	HC.Y	0.391	0.358	0.426				CD.Y	0.592	0.455	0.738			
*R*	2017	Derived	HC.Y	0.340	0.301	0.377				CD.Y	0.362	0.213	0.517			
*R*	2018	Derived	HC.Y	0.321	0.285	0.357				CD.Y	0.391	0.238	0.536			
*R*	2019	Derived	HC.Y	0.485	0.447	0.523				CD.Y	0.573	0.405	0.744			
*R*_2_		Derived	HC	0.313	0.271	0.358										
*p*		Fitted								CD.Y	0.886	0.875	0.897	0.877		
*q*		Fitted								CD.Y	0.022	0.018	0.027	0.019		
*β*_D_		Fitted								CD.Y	64.349	44.279	85.345	81.275	1.000	Inf

*Note*: See Table A1 for parameter names. Acronyms are defined as follows. EV, expected value; LCL & UCL, lower and upper credible limits; LCL_0_, one‐sided LCL; Pr, probability; LER, log evidence ratio; MAD, mean absolute difference; maxR, maximum ratio; meanR, mean ratio; *R*_2_, recruitment to age 2.
